# Abdominal Hydatidosis: Unusual and Usual Locations in a North Indian Population

**DOI:** 10.7759/cureus.4380

**Published:** 2019-04-03

**Authors:** Shreshtha Jain, Sachin Khanduri, Umar F Sagar, Poonam Yadav, Mushahid Husain, Tariq Imam

**Affiliations:** 1 Radiology, Era's Lucknow Medical College and Hospital, Lucknow, IND; 2 Radiology, Era’s Lucknow Medical College and Hospital, Lucknow, IND

**Keywords:** hydatid cyst, abdomen, indian population, unusual locations

## Abstract

Objective

The objective of this study was to assess various locations in the abdomen wherein hydatid cysts can occur in an Indian population.

Materials and methods

A retrospective study was conducted on 38 patients of 20-55 years of age in an Indian population, who were diagnosed with hydatidosis on ultrasound and computed tomography. The measurement and location of the cysts were taken by the double operator method. Patients were followed up until post-surgical and histopathological diagnosis.

Results

Among the observed patients, the most common age group was 30-40 years (36.85%), with male predominance (76%). The most commonly involved organ was liver (71.1%) followed by the kidney (10.5%) and peritoneum (8%), pancreas (2.6%), spleen (2.6%), common bile duct (2.6%) and adnexa (2.6%).

Conclusion

In spite of the usual presenting features, the locations of the cysts were unusual, thus warranting the importance of making the diagnosis before rupture of the cysts and thereby preventing life-threatening complications such as anaphylactic shock.

## Introduction

Hydatid disease is a zoonotic disease caused by the larvae of the cestode species of the genus *Echinococcus,* which includes *E. granulosus*, *E. multilocularis*, *E. vogeli,* and *E. oligarthus* [1]. Classical cystic echinococcosis (CE) is caused by *E. granulosus* complex. *E. multilocularis*is is responsible for alveolar echinococcosis, and *E. vogeli* causes polycystic echinococcosis [2].

CE has variable clinical presentations, ranging from asymptomatic diseases to acute emergencies. Hydatid cysts have been reported in various body organs such as the liver, lungs, peritoneum, bones, ovaries, breast, and the brain. Small, intact cysts have no specific characteristic symptoms. Clinical manifestations of this disease depend on multiple factors such as the organ involved, the site and size of the cyst, the interaction between the expanding cysts and the adjacent organ structures, complications related to cyst rupture, the spread of protoscoleces, and bacterial infections. In patients with infection or cyst rupture, the presentation may be severe [3-10].

Hydatid cysts most commonly involve the liver (in 59% to 75% of cases), followed by the lung (27%). Involvement of the kidney (3%), bone (1% to 4%), and brain (1% to 2%) are not so common. Other sites such as the heart, spleen, pancreas, and voluntary muscle tissue are very rarely involved, but no site is immune [11-13].

The involvement of abdominal organs (e.g., the liver) is the most common, and therefore, most studies have been dedicated to hepatic hydatidosis or whole-body hydatidosis. However, scant literature is available regarding its presence in other abdominal organs like the pancreas, spleen, kidneys, peritoneum, common bile duct, and adnexa. Most cysts in these areas were unruptured and removed whole. To enhance our understanding of hydatid disease in unusual abdominal locations, we conducted this study in an Indian population to assess various locations in the abdomen wherein hydatid cyst can occur.

## Materials and methods

We conducted this retrospective study of patients aged 20 to 55 years at Era’s Lucknow Medical College and Hospital in Lucknow, India from June 2016 to June 2018. The study included 38 patients who underwent ultrasonography and computed tomography (CT) at the radiology department of the hospital and were diagnosed with hydatidosis. Data collected included demographic information, clinical features, and laboratory findings. Patients allergic to contrast media, those with deranged renal functions, pregnant females, patients younger than 20 years, and patients not consenting for contrast study and surgical procedures were excluded from the study.

We located and measured the cysts using a double operator method. Axial and sagittal CT images were obtained, and measurements were taken accordingly. Patients were monitored via follow-up until the final post-surgical histopathological diagnosis of hydatid cyst.

## Results

**Figure 1:**
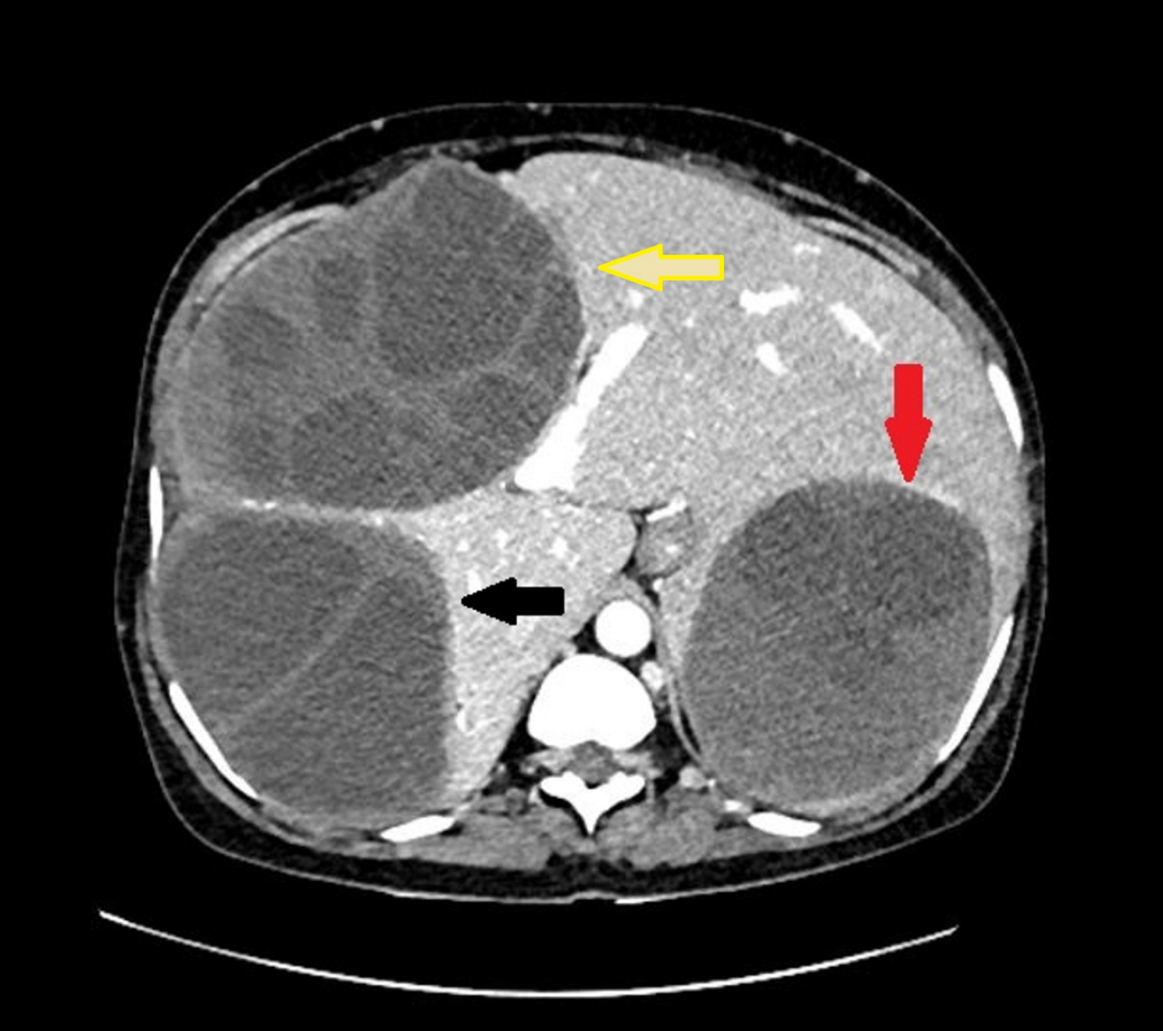
Hydatid cyst in the liver and peritoneum on CT CECT abdomen axial image showing a large, well-defined hypodense lesion in the right lobe of the liver (black arrow) with mildly enhancing wall and internal septations. Similar hypodense lesion is seen in the left lobe of the liver (red arrow) with debris within and no septations. Another moderate-sized, well-defined, rounded cystic lesion with mildly enhancing walls and internal septations is seen in the peritoneum (yellow arrow) with scalloping of the adjacent liver margins. CECT: contrast-enhanced computed tomography, CT: computed tomography

**Figure 2:**
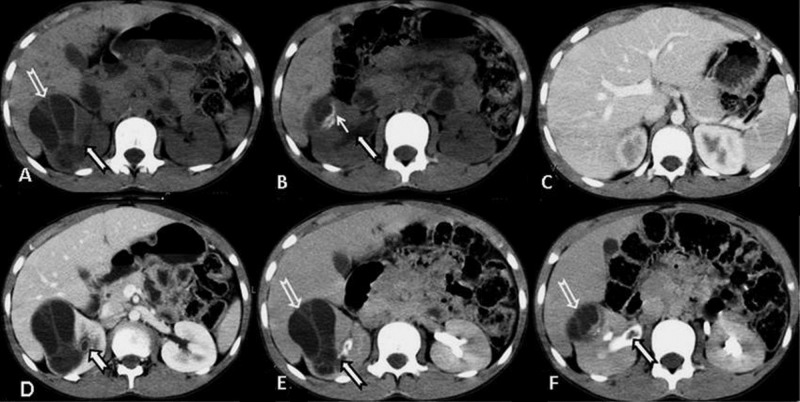
Hydatid cyst in the kidney on CT Axial unenhanced (A and B), nephrographic (C and D), and excretory phase (E and F) images from a CT urogram demonstrate a moderate-sized, non-enhancing, low-attenuation,well-marginated, multi-septated cystic mass in the right kidney involving the outer cortex anterior to the renal pelvis (open white arrow) with peripheral calcifications dorsally (thin white arrow). Extension of the daughter cysts is noted in the right pelvicalyceal system extending into the right ureter which is suggestive of the rupture of the parent cyst (white arrow with black outline). CT: computed tomography

**Figure 3:**
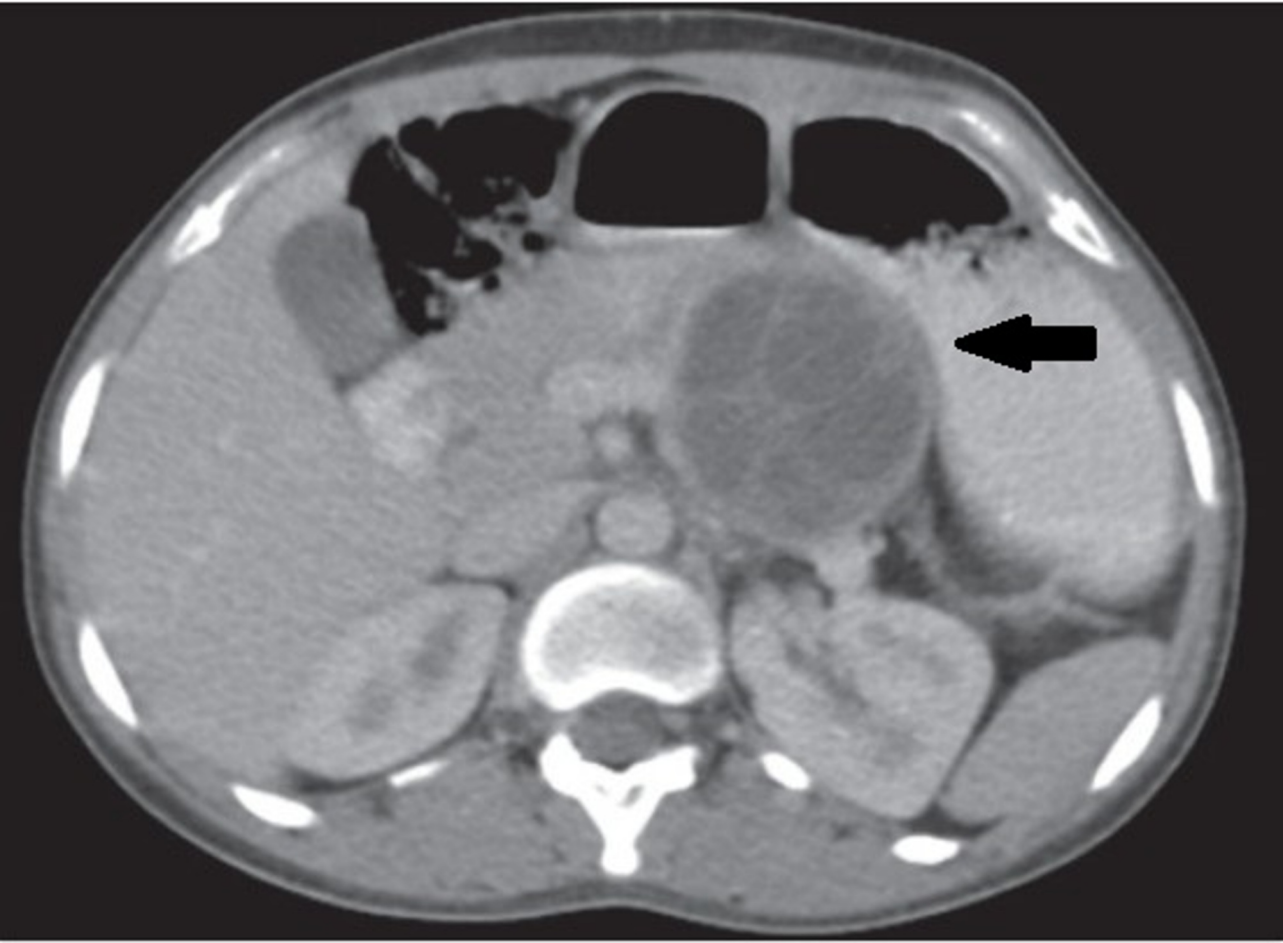
Hydatid cyst in pancreas on CT Axial CECT image shows a moderate–sized, well-defined, lobulated, thin, mildly enhancing walled intrapancreatic cystic lesion in the distal body and tail region of the pancreas (black arrow). The lesion shows multiple, internal, well-defined cystic areas/daughter cyst/septations, with no evidence of calcification. Mass effect is seen on the spleno-pancreatic axis, and the splenic vein is compressed and narrowed. CECT: contrast-enhanced computed tomography, CT: computed tomography

**Figure 4:**
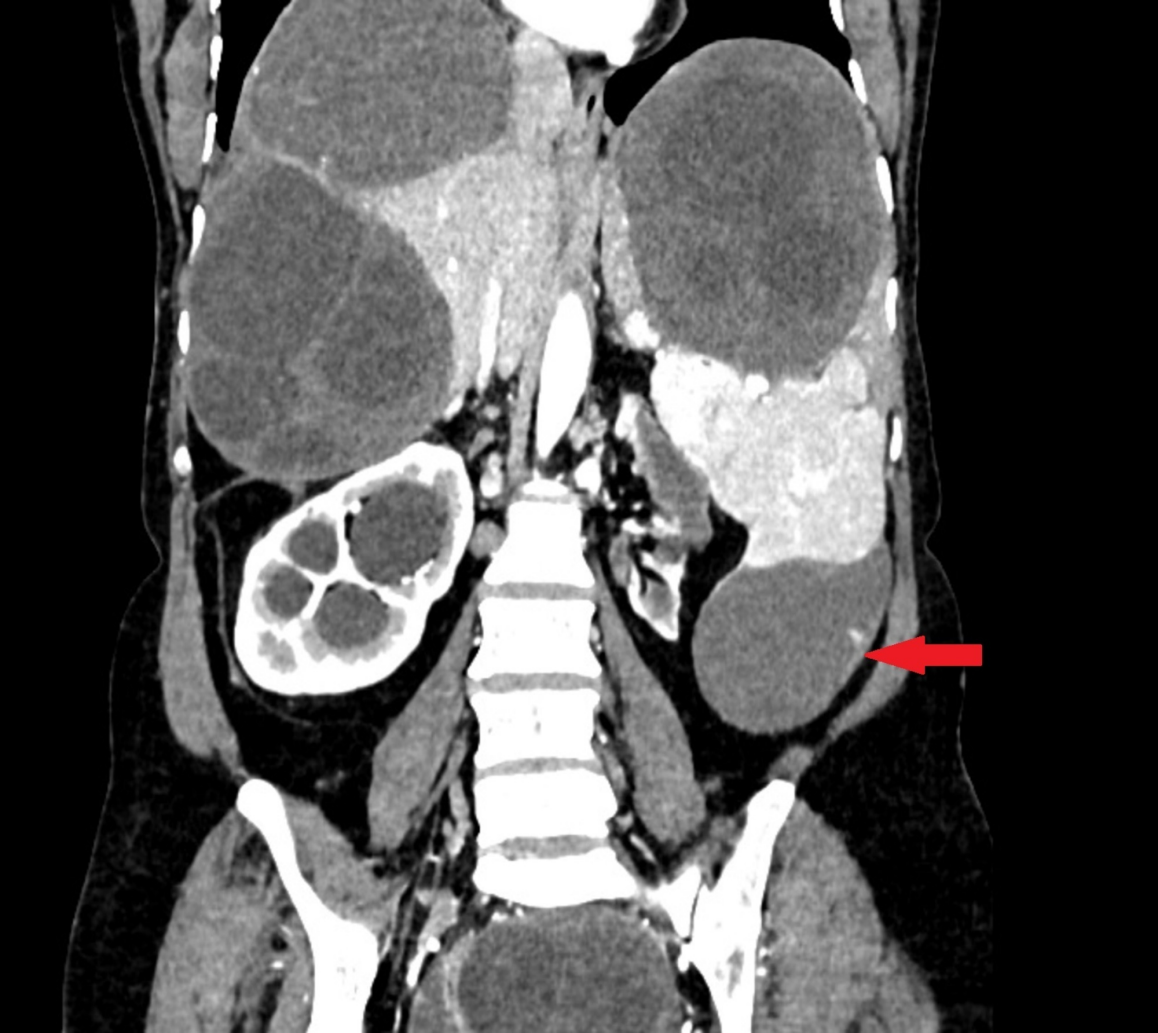
Splenic hydatid cyst on CT CECT abdomen coronal image shows a moderate-sized, well-defined, rounded cystic lesion in the spleen (red arrow) with septation. CECT: contrast-enhanced computed tomography, CT: computed tomography

**Figure 5:**
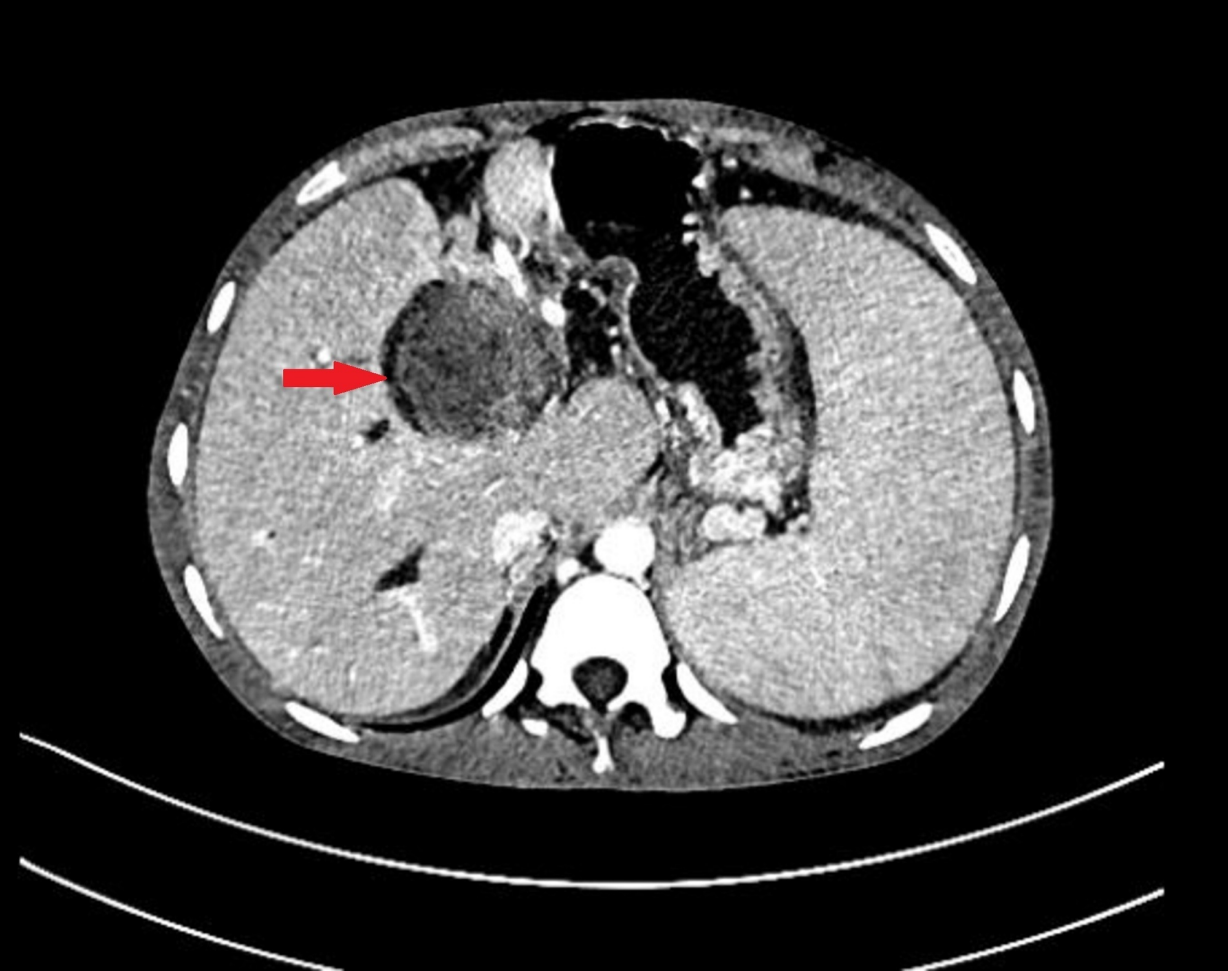
Hydatid cyst in the common bile duct on CT CECT abdomen axial image shows a well-defined rounded heterogeneously hyperdense lesion (red arrow) with peripheral calcification at porta. CECT: contrast-enhanced computed tomography, CT: computed tomography

**Figure 6:**
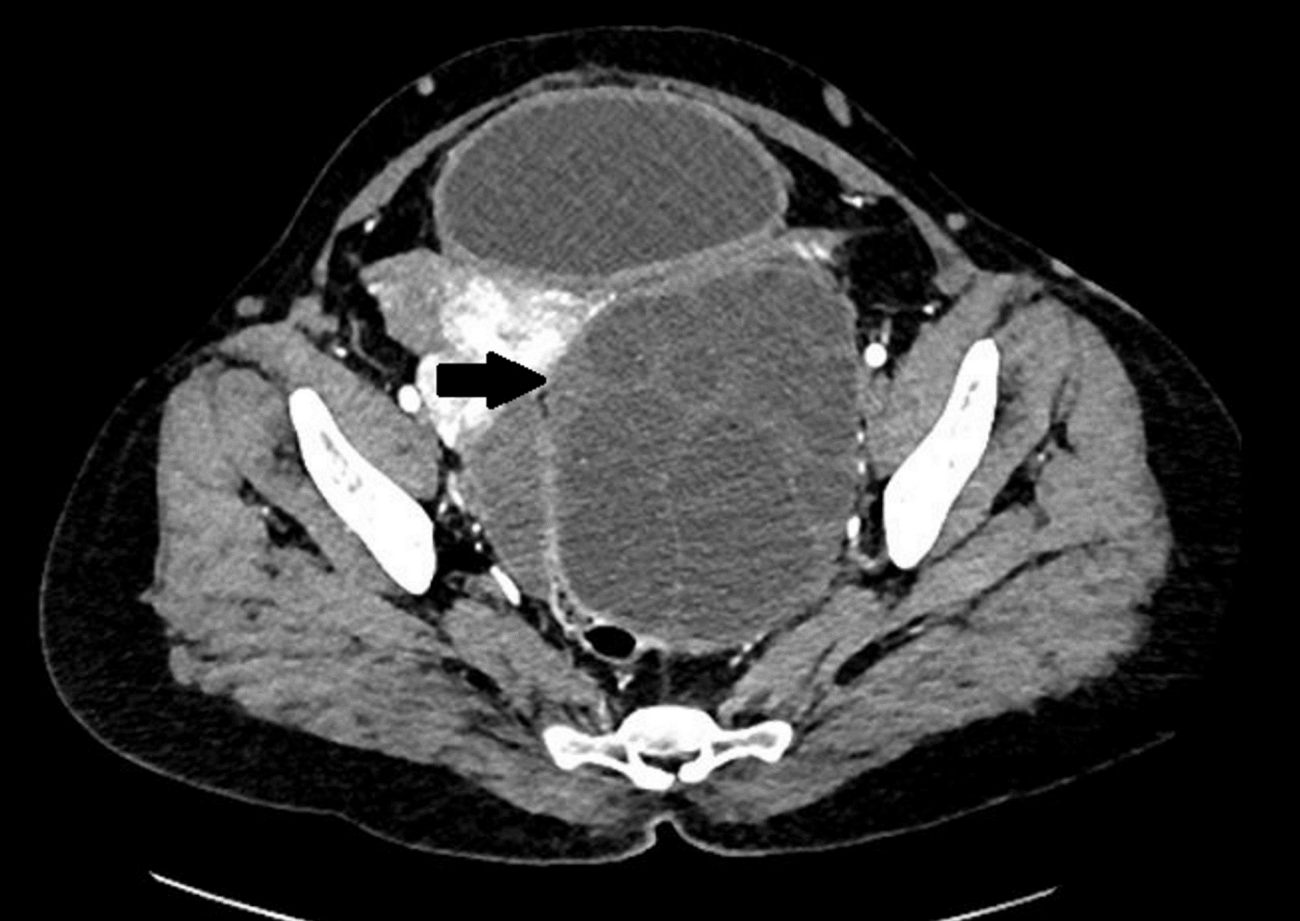
Adnexal hydatid cyst on CT CECT abdomen axial image shows a large, well-defined, rounded, thin-walled, multiloculated cystic lesion in the pelvic cavity on the left side. Left ovary is not visualized separately. CECT: contrast-enhanced computed tomography, CT: computed tomography

Thirty-eight patients were included in our study (Table 1), of whom 29 were men and nine were women. Being a tertiary care center, most patients came to our hospital from rural areas presenting with abdominal pain, abdominal lump, fever, and vomiting (Table 2). Two patients presented with concerns of obstructive jaundice as itching and yellowish discoloration of the eyes. The liver was the most commonly involved organ (Figure 1; 71.1%), followed by the kidney (Figure 2; 10.5%) and the peritoneum (Figure 1; 8%) pancreas (Figure 3; 2.6%), spleen (Figure 4; 2.6%), common bile duct (Figure 5; 2.6%), and adnexa (Figure 6; 2.6%), as shown in Table 3.

**Table 1:** Division of patients according to age

Age Group	Number of Patients	Percentage
20–30	12	31.58%
31–40	14	36.85%
41–50	9	23.68%
51–55	3	7.89%

**Table 2:** Presenting concerns

Presenting Concern	Number of Cases
Pain in the abdomen	27
Abdominal lump	15
Fever	5
Vomiting	8
Jaundice	2

**Table 3:** Location of hydatid cysts

Location	Number of patients	Percentage	95% CI	
			Lower	Upper
Liver	27	71.1	56.7	85.5
Kidney	4	10.5	0.8	20.2
Peritoneum	3	8	0.0	16.6
Pancreas	1	2.6	0.0	7.7
Spleen	1	2.6	0.0	7.7
Adnexa	1	2.6	0.0	7.7
Common bile duct	1	2.6	0.0	7.7

## Discussion

Hydatid disease is a unique parasitic disease endemic in many parts of the world. The parasite is echinococcus and has two hosts; the primary host is a carnivore such as a dog. Once the echinococcus cyst reaches the human liver, they grow to 1 cm in six months and then 2 to 3 cm annually thereafter, depending on the host’s tissue resistance [14-16]. The liver is the first line of defense in the human body and therefore becomes the first organ to get involved before the cyst involves the other organs. The disease commonly involves the lungs and liver. However, unusual locations like the spleen, kidney, common bile duct, peritoneum, adnexa, and head of the pancreas have been reported [17-19].

Our study shows various unusual locations of abdominal hydatidosis in north Indian population and their clinical scenario and common features. Most of the patients in our study were aged 30 to 40 years, with a male predominance from rural communities of low socioeconomic status. The most common presenting feature in our cases was abdominal pain with lump. However, hydatid of the common bile duct and pancreas presented with the features of obstructive jaundice, and hydatid of the kidneys presented with the passage of small rounded white grape like structures in the urine.

Our study showed that, despite the usual presenting features, the locations of the cysts were unusual and warranted further investigation in the disease. Radiological studies of such cases also need more emphasis on commonly ignored locations such as the common bile duct, adnexa, and peritoneum, so that the diagnosis can be made before the cysts rupture, thereby preventing the patient from life-threatening complications such as anaphylactic shock. One of our cases illustrated timely radiological diagnosis of a hydatid cyst found in the common bile duct led to prompt surgical removal of the cyst, intact, and unruptured.

Our study has some notable limitations. Other studies on hydatid cysts have used various serological tests such as immunoelectrophoresis, enzyme-linked immunosorbent assay, latex agglutination, indirect hemagglutination, and polymerase chain reaction for diagnosing along with species specification of Echinococcus. We did not conduct these tests for species specification of Echinococcus, and we were unable to draw any relationship between different species of Echinococcus and other species more commonly responsible for hydatid cyst at usual and unusual locations in the abdomen. 

## Conclusions

Hydatid cysts can form in unusual, varied locations. Radiological studies of such cases should include less common locations such as the common bile duct, adnexa, and peritoneum, so that the diagnosis can be made before the cyst ruptures, preventing the patient from developing life-threatening complications like anaphylactic shock.
